# What can art history offer medical humanities?

**DOI:** 10.1136/medhum-2023-012763

**Published:** 2024-05-30

**Authors:** Suzannah Biernoff, Fiona Johnstone

**Affiliations:** 1 School of Historical Studies, Birkbeck University of London, London, UK; 2 Institute for Medical Humanities, Durham University, Durham, UK

**Keywords:** art and medicine, art history, COVID-19, Medical humanities, Art

## Abstract

This article charts the emergence of visual medical humanities as a space of academic research, creative practice and lively critical debate, with a focus on how art historical scholarship has influenced the field’s formation. Concentrating on developments over the past decade, it offers an overview of current scholarship while highlighting opportunities and challenges for the future. We begin with a survey of medical and health humanities handbooks and readers, noting that their engagement with art and visual culture is predominately limited to the contexts of therapy, clinical pedagogy and medical history. The main part of the article explores art historical scholarship in relation to three areas of significance for the medical humanities. First, we address art historical research that engages with medical history, identifying major *topoi* including the anatomical body, the doctor-patient encounter and the close relationship between clinical and artistic vision; we argue that this work has tended to presume, rather than explicitly articulate, its relationship to medical humanities and recommend that art historians wishing to engage more deeply with the medical humanities need to clearly communicate what their work brings to wider debates in the field. Second, we explore contemporary arts practices that mobilise health-related experiences, forms of care and practical activism: medical humanities, we argue, has much to gain from a critical engagement with contemporary (as well as historical) art. Third, we review three art history-led projects that are redefining the field and promoting new models for collaborative ‘entanglement’ across disciplines: *Art HX: Visual and Medical Legacies of British Colonialism; Visualizing the Virus*; and *Confabulations: Art Practice, Art History, Critical Medical Humanities*. By arguing for the vital importance of attending to the critical complexities of art and visual culture, this article aims to enrich existing debates and provoke a new wave of visually engaged medical humanities scholarship.

## Introduction

In their introduction to *The Edinburgh Companion to the Critical Medical Humanities*, Anne Whitehead and Angela Woods note that literary, philosophical and historical approaches have had more influence within medical humanities than visual studies (2016, 19). Writing in *Medicine, Health and the Arts: Approaches to the Medical Humanities*, Ludmilla Jordanova observes a ‘surprising fragmentation’ between scholarship on medicine and the visual arts, with each field producing a distinct specialised literature that appears ‘unconnected in terms of themes, sources, theories, approaches, guiding assumptions and target audiences’ (2014, 43). Since the publication of these two texts, a range of interventions by scholars and creative practitioners have helped to fashion a more productive and critically engaged relationship between medical humanities and art history and visual culture. This article maps those developments and charts the formation of the emergent field of visual medical humanities, an area of research and practice concerned with visual and material culture as it relates to structures and experiences of health, illness, intervention and care. Through this mapping, the article expands on Johnstone’s ‘Manifesto for a Visual Medical Humanities’ (2018), which argued that the role of art and visual culture within medical humanities had too frequently been reduced to the illustrative (as a way of making biomedical research more ‘accessible’ to a broader public) or instrumental (in the service of producing more ‘empathetic’ doctors), and made the case for a more critically engaged approach that would pay attention to the affective and embodied dimensions of visual experience, embrace the productive ambiguity of visual artefacts and interrogate the expectations routinely placed on certain visual objects to do particular types of jobs. By showing how scholars of art history and visual culture have engaged with issues that are fundamental to medical humanities over the past decade, and by identifying areas for further development, this article advocates for an enhanced critical integration of art historical methods and materials in medical humanities research, pedagogy and practice.

We approach the discipline of art history in its most expanded sense as inclusive of visual culture; that is, as a discipline that does not confine itself to addressing the kinds of objects normatively recognised as ‘art works’, but which applies the methods of art history to a vast range of visual materials.^1^ For the purposes of medical humanities, this might include medical illustration and imaging; public health posters, films, infographics and other visual health communication materials; films and television programmes with health-related themes; visual social media; the collections of medical museums and archives; art therapy and other therapeutic uses of art; graphic medicine; the use of the visual arts in clinical education and so on.^2^ This list is not exhaustive. Given the wealth of visual materials that might potentially be addressed, the title of this article, ‘What can art history offer medical humanities?’ may seem somewhat over-ambitious. However, since Jordanova’s 2014 essay ‘Medicine and the Visual Arts,’ there has been no single text or other publication that has advocated for the contributions that art history might make to medical humanities. This article, therefore, aims to offer an accessible overview of current scholarship, while identifying opportunities for a deeper and more critical engagement between the two fields. We hope it will be of use to medical humanities researchers working in humanities disciplines that do not typically centre visual culture, but who are curious as to how working with artworks and other visual materials might enhance their own scholarship; to practitioners in the visual arts seeking to locate their own practice in relation to current developments; and to students on art history and visual culture courses as well as medical humanities programmes (recognising that many universities are now seeking to incorporate more visual materials into taught medical humanities courses).

An article like this one is by necessity an act of selection: as a starting point, we have chosen to centre scholarship by researchers working within and alongside art history (as opposed to scholarship on visual culture produced by social scientists or literary specialists); this is not because we believe that art historians have the monopoly on writing about visual culture, but because we want to explore the specific affordances of our own discipline in relation to critical medical humanities research. Within this, we have concentrated on texts and projects that are either indicative of the general direction of scholarship or which bring art history and medical humanities together in particular innovative and exciting ways. Finally, we have focused predominantly on work produced by scholars based in the UK, the USA and Canada, partly because this is the scholarship that we are most familiar with and also to give geographical focus to an already ambitiously wide-ranging enquiry. Our hope is that other scholars will find this article a helpful springboard for research that extends beyond these pragmatic parameters.

Two particularly significant forces have influenced the most recent work in this field: the COVID-19 pandemic, and the increasingly pressing task of addressing the racial and geographical biases that have made art history a fundamentally Eurocentric and colonialist discipline from the eighteenth century onwards (see Grant and Price 2020; Flores, Martín, and Black 2024). Current calls to decolonise art history build on the postcolonialist, Marxist, feminist and queer theories and methods incorporated by the discipline from the 1970s onwards. Giving new critical urgency to these approaches, the COVID-19 pandemic made painfully evident the uneven distribution of health inequalities in relation to race, disability, social class, education, income and gender. Contemporary art writing was quick to address the questions raised by the pandemic in an imaginative and provocative manner, while art historians drew parallels between the visual culture of COVID-19 and that of previous pandemics including cholera, the 1918 influenza epidemic and HIV and AIDS.^3^ Essays such as Sria Chatterjee’s ‘Contingent Contagion’ (2022) used visual media to consider the politics of visibility raised by the pandemic: through a close reading of images produced in response to COVID-19 and other pandemics by artists in India and Australia, Chatterjee explored a range of complex issues including vaccine imperialism and xenophobia, emphasising the indispensability of visual studies approaches not only as critique but as ‘the first revolutionary step towards action in a world where much of life and its politics is invisible’ (2022, 12). Chatterjee set up the digital project *Visualizing the Virus* in 2020; this and other recent art history-led interdisciplinary medical humanities projects, including *Art HX: Visual and Medical Legacies of British Colonialism* and *Confabulations: Art Practice, Art History, Critical Medical Humanities* are discussed in the final section of this article.

We begin this article by surveying a range of medical and health humanities readers and handbooks and offering a summary of how medical humanities has engaged with art history and visual culture to-date. Supplemented with a short genealogy of medical humanities as a field, this allows us to identify how and why medical humanities has so often approached art and visual culture in a primarily therapeutic or pedagogic mode, or alternatively, through the lens of medical history. We then turn to recent art historical scholarship that engages with the history of medicine and medical humanities, identifying common themes and tropes, including an emphasis on anatomical imagery and doctor and patient identities and relationships. In the middle section of the article, we look at contemporary art history writing on the aesthetics of chronic illness and care, which extends its line of enquiry beyond the explicitly medical sites of the anatomy theatre and the clinical consulting room to draw attention to the broader social, cultural and political contexts of health. We propose that contemporary art projects—which often take place outside of clinical or university-led medical humanities spaces—frequently engage with questions of experience and practical activism that resonate deeply with the intellectual, ethical and aesthetic values of the medical humanities. Finally, we look at a range of interdisciplinary art history-led medical humanities projects that are currently in development or working towards publications, to give a sense of the future direction of the field. We conclude by arguing for the vital necessity of an ongoing critical engagement with art and visual culture (both past and present) within medical humanities scholarship and practice.

## The visual arts in medical humanities

A survey of medical and health humanities handbooks and readers published over the past decade offers a view of how the field has engaged with art history and its methods. *Medicine, Health and the Arts*, edited by Victoria Bates, Alan Bleakley and Sam Goodman (2013), includes Ludmilla Jordanova’s valuable if necessarily brief survey of medicine and the visual arts in the post-war period in Britain; Jordanova concedes that the visual arts deserve ‘a much fuller treatment from a medical humanities perspective than has been possible here’ (2014, 62). The communicative and therapeutic qualities of art, as well as its potential for enhancing empathy in medical practitioners and for engaging the public, are foregrounded by Victoria Tischler in entries published in *Health Humanities*, edited by Paul Crawford (2015), and the *Routledge Companion to Health Humanities*, edited by Paul Crawford, Brian J. Brown, and Andrea Charise (2020). Intriguingly, Tischler suggests that the potential therapeutic benefits of visual art have not yet been fully explored by medical humanities precisely because ‘traditionally, artistic and cultural artefacts have been reviewed, researched and curated by art historians and cultural theorists’ (2015, 107); this hints at the perceived limitations of art history from the perspective of the social sciences. Essays by Sander Gilman in the *Health Humanities Reader*, edited by Therese Jones, Delese Wear and Lester D. Friedman (2015), and Suzannah Biernoff in *The Edinburgh Companion to the Critical Medical Humanities*, edited by Whitehead and Woods (2016), attend to medical histories of visuality and the challenges of representing the usually-invisible phenomena of pain pictorially. Also in *The Edinburgh Companion,* essays by Edward Juler (2016) on surrealist anatomical images and artist Rachael Allen (2016) on drawing in the dissection theatre both build on a longstanding relationship between art history and anatomical representation.

The *Routledge Handbook of the Medical Humanities*, edited by Alan Bleakley (2019), contains chapters on the performing arts (Hooker and Dalton 2019; Wilson, Brett-Maclean, and Eacott 2019; O’Brien and Bouchard 2019) as well as on artists’ books (Bolaki 2019), gathered together under the section sub-heading of ‘Medicine as performance and public engagement.’ The *Research Methods in Health Humanities* handbook, edited by Craig M. Klugman and Erin Gentry Lamb (2019), includes a chapter on ‘Art History’ by Siobhan Conaty, which emphasises art history’s methods of close looking, contextualising, formal analysis and critical thinking about visual materials; Conaty (who has written extensively on art history as a method for the medical humanities) asserts that ‘art history methods are indeed health history methods’ (2019, 110) and offers a useful reminder that the methods of art history are not limiting to looking at what might strictly be considered as artworks (Conaty suggests that they might be applied to public health murals). At a glance, this survey tells us that the material that art history might (and does) address in relation to medical humanities is potentially vast, if currently underutilised.^4^

At this point, it is helpful to consider a brief genealogy of medical humanities as a field. Alan Bleakley identifies three developmental strands for medical humanities in the UK (as Bleakley notes, medical humanities in the USA and Canada follow a slightly different trajectory): arts-for-health (which develops out of the growth in arts therapies after the Second World War); the role of the Wellcome Trust in establishing the history of medicine as a well-funded discipline; and medical humanities approaches in medical education (Bleakley 2013, 20). This history gives some clues as to why the medical and health humanities have tended to approach visual culture as predominantly therapeutic and/or pedagogic (eg, [Bibr R75][Bibr R114]); as part of scholarship in the history of medicine ([Bibr R37][Bibr R38]); or in relation to medical education (Hooker and Dalton 2019; Wilson, Brett-Maclean, and Eacott 2019; O’Brien and Bouchard 2019). Acknowledging ongoing debates about the risks of the instrumentalisation of humanities disciplines in medical humanities, we have deliberately chosen not to address the use of art and visual culture in arts-for-health or in medical education and clinical practice in this present text. Instead, we focus specifically on how art history (and art history adjacent) scholarship and practice has intersected with the research-focused agendas of critical medical humanities.

## Art history and the history of medicine

Art historians have documented the cross-fertilisations of art and medicine since the Middle Ages, often with a particular focus on the visual dynamics of the patient–doctor relationship and the spectacle of the anatomy theatre.^5^ The clinical encounter has been identified as the ‘primal scene’ of the medical humanities by Whitehead and Woods (2016, 2), so it is perhaps unsurprising that this features heavily in art history writing on medicine. The June 2013 special issue of *Medical Humanities* on patients’ portraits, edited by Jordanova, is one such example, highlighting the similarities between portrait sittings and the doctor–patient encounter. Both relationships rely on ‘close visual scrutiny.’ Both involve the exercise of power and the negotiation of social conventions (Jordanova 2013, 2). All portraits, arguably, enact a diagnostic gaze that looks for meaning in particularity while transforming individuals into types. Articles by Keren Hammerschlag, Susan Sidlauskas, Douglas James, Natasha Ruiz-Gómez and Tania Woloshyn explore these affinities between the clinical and the artistic gaze through case studies ranging chronologically from the eighteenth century to the 1940s. One of the themes running through their essays is the close proximity of art and medicine, artists and physicians, at least until the professionalisation of medical illustration during and after the Second World War (Sawchuk 2012; Sappol 2017).^6^ In her article on the sculptures of pathological specimens created by Doctor Paul Richer at the Hôpital de la Salpêtrière in Paris in the 1890s, Ruiz-Gómez elaborates on a new category of medical specimen: the so-called ‘scientific artwork’ (2013, 4). Douglas James, discussing John Hunter’s clinical practice in Georgian London, makes a similar case for understanding medical representations as ‘incarnations of medical skills and medical knowledge’ (2013, 11).

Mary Hunter’s *The Face of Medicine: Visualising Medical Masculinities in late Nineteenth-Century Paris* (2016) uses the iconography of three medical men—Louis Pasteur, Jean Martin Charcot and Émile Péan—to chart the cross-currents flowing between the two disciplines. The ‘reflexive relationship between art and medicine’ across the long nineteenth century is also the organising theme of Anthea Callen’s *Looking at Men: Anatomy, Masculinity and the Modern Male Body* (2018, 11) and the point of departure for Biernoff’s writing on the aesthetics of disfigurement, which begins where Callen ends: with Henry Tonks’ delicate pastel studies of First World War servicemen with facial injuries ([Bibr R11][Bibr R13]). Medical art takes on another role in these intimate and difficult drawings: not just ‘help[ing] medicine visualise its normal and its pathological bodies’ (Callen 2018, 13) but registering vulnerability, trauma and stoicism. Contact is also a recurring motif in Mechthild Fend’s work, which links the popularity of dermatological wax moulages in nineteenth-century France to the invention and scientific application of photography (also a contact medium involving a photosensitive medium being ‘touched’ by light). What all of this research has in common is the conviction, to quote Fend, that medical/artistic artefacts are ‘complex objects’ (2022, 41). Understanding how and why they were made and used involves painstaking research, but these objects are complex in an emotional sense too. Wax moulages of skin diseases and life drawings of hospital patients connect us, tangibly, to people who were ill or in pain. This makes them ‘troubling objects’, not just for their original makers and users, but for the scholars who work with them (Fend 2022, 41; Biernoff 2017).

Fend’s observation is echoed in much of the recent historical scholarship in the visual medical humanities, which insinuates the writer’s body into the scholarly text. ‘It is hard to look at them without getting one’s own body involved, without the sensation of an itch or the feeling of disgust’, she writes of her dermatological waxes, with their blisters and encrustations (2022, 26). Historical studies of clinical photography have been similarly ‘troubled’ by sensations and affects. Self-reflexivity serves, in this academic corpus, to foreground the bodily and historically situated conditions of viewing (and of scientific/historical knowledge), but it is also a way of attending to the bodies and lives whose traces persist, mutely, in archives and museums (Bate 2021; Pichel 2017; [Bibr R87][Bibr R88]; Rose 2000; Slobogin 2022).

Self-reflexivity and a concomitant attentiveness to affective experience also characterise a number of recent interventions that explore the gendered dynamics of clinical medicine in relation to women’s lived experiences of illness. Revisiting Edvard Munch’s experimental lithographic prints of ‘sick girls’ through the interplay of ‘observational and diagnostic impulses’ and ‘affective experience,’ Allison Morehead articulates the value of these works in offering the present-day viewer ‘pathways to thinking and feeling critically about medicine’ (2022, 53, 59). Adopting a deliberately transhistorical perspective, Morehead argues that these artworks respond ‘*avant la lettre* to Woolf’s calls for a “new language”, “more primitive, more sensual, more obscene”, that could represent the experiences of being ill’ (Morehead 2022, 65; citing Woolf 1926, 7). Working in a similarly transhistorical and affective register, Gemma Blackshaw and Alice Butler’s current project ‘Sick Women Correspondents’ uses letter writing as a creative and embodied research method to reconsider the cross-historical experiences of two ‘sick women’, the nineteenth-century dancer, singer and TB tourist Bessie Bruce; and the dancer, actress and writer Cookie Mueller, who died from causes related to AIDS in 1989 (Blackshaw and Butler 2023).

As noted above, with a few exceptions and until fairly recently, much of this literature on the visual cultures of medicine had focused on doctors (usually white and male) and their patients, and on the contexts of clinical medicine and medical training, with a particular emphasis on the anatomical body. The same generalisation has been true of medical humanities as a field, which until recently emphasised the clinical encounter and treatment or recovery narratives over the many non-medical and biopolitical dimensions of health and illness. In their essay in the *Edinburgh Companion to the Critical Medical Humanities*, Des Fitzgerald and Felicity Callard list the ‘favoured topoi’ of medical humanities scholarship: ‘the suffering patient, a doctor’s practice of clinical care, the exemplary site of the clinic, and cancer’ (2016, 41). Yet these ‘overly resonant configurations’ are not predetermined or inevitable. ‘What if the task of the medical humanities were to encourage the emergence of different topoi,’ they ask. ‘What if illness were not imagined, for example, as co-located with or coincidental to a body?’ (2016, 48).

There are signs that just such a recalibration is underway. Coinciding with debates about what it might mean to decolonise art history (Grant and Price 2020; Flores, Martín, and Black 2024) there has been new attention to the racial, colonial, and environmental subtexts of western medicine, its visual traditions and material cultures. Many of these interventions decentre the individual patient or doctor and instead take the form of object biographies, tracing the geographical movement of human remains (Au 2017; MacDonald 2017); public health exhibitions (Kenny 2017); or art objects ([Bibr R44][Bibr R45]). Hammerschlag’s piece for the Objects in Motion series in *British Art Studies* attempts, in a scholarly act of ‘re-skinning,’ to restore the racial identities of cadavers dissected by Joseph Maclise for his *Surgical Anatomy*. On discovering that two plates featuring a black man in the 1851 British edition were missing entirely from later American editions of the atlas, she reconstructs the circumstances of this ‘transatlantic erasure’ (2021).

In Ari Larissa Heinrich’s *Chinese Surplus* (2018), bodies are ‘diasporic’ entities: alienated from their original owner-custodians and subjected to extraction, dissection, preservation, exhibition, transplantation, transportation and global exchange. His case studies range from literary and cinematic portrayals of organ harvesting and transplantation, to the ‘cadaver group’ artists who staged guerrilla-style installations in Beijing in the 1990s and 2000s, to discussions (in China, Hong Kong, Taiwan and elsewhere) about the ethics of Gunther von Hagens’ travelling *Body Worlds* exhibition with its plastinated cadavers rumoured to be sourced from Chinese prisons. Heinrich’s aim is not to uncover the truth (or otherwise) of the rumours about *Body Worlds*, or the extent of the organ trade in the authoritarian market economy that has transformed post-Mao China. As a cultural historian, he is interested in how Chinese bodies are imagined and represented in a biotechnological age: as object, as commodity, and as surplus.

Anna Arabindan-Kesson’s article ‘Transmission and Transfer: Plantation Imagery and Medical Management in the British Empire,’ published in *Art History* in 2022, offers a third example of art historical scholarship that brings decolonial frameworks to bear on visual histories of medicine. Addressing visual constructions of Caribbean plantations through the art historical tradition of the ‘picturesque’, Arabindan-Kesson shows how the aesthetics of landscape mediated medicalised knowledge about colonial space and colonised people through a visual logic of extraction, observation and control. Noting that ‘the history of art and the history of medicine share a reliance on visual acuity’, Arabindan-Kesson argues that this reliance ‘coalesced on the plantation to produce ways of seeing and relating to people and the environment, that continue to shape the ways we care for, and treat, each other today’ (2022, 490). Drawing on Stuart Hall’s dialogic methodology of looking *across* or *between* (rather than *with* or *through*), Arabindan-Kesson foregrounds attentiveness to the ‘epistemological implications’ of different ways of seeing (2022, 475), and advocates for close engagement with visual materials—both historical objects and contemporary artworks—to consciously ‘re-orientate our sightlines’ (2022, 477).

This section has outlined a body of scholarship that places art history in dialogue with medical history. Curiously, however, many of these scholars do not explicitly place their work in conversation with current thematic debates, theoretical frameworks or methodological developments in critical medical humanities: in many cases, the relevance of art historical scholarship on anatomical bodies or clinical identities (to cite the two major *topoi*) to medical humanities is presumed rather than expressly articulated. Additionally, much of the careful art historical work that could be productively brought to bear on medical humanities scholarship remains siloed within discipline-specific publications, including specialist journals and exhibition catalogues (which can be particularly inaccessible, being rarely digitised and often produced in fairly limited print runs). This represents both a challenge and an opportunity for art historians to clearly articulate what their work brings to wider debates in the field (rather than assuming that this is obvious).

## Crip aesthetics, care and ‘aesthetic refusal’

Contemporary art has taken a ‘turn to health,’ as the curator Rodríguez Muñoz (2020, 13) observes in the introduction to the collected volume *Health (Documents of Contemporary Art*), with recent art history writing on the aesthetics of chronic illness and care reflecting a reorientation towards the wider biopolitics (and environmental contexts) of health and well-being. In her 2021 article ‘Chronic illness as critique,’ Giulia Smith identifies a tendency in artistic practice over the past decade to present sickness not as an individual experience, but as a symptom or metaphor of collective crisis. Although the article was written before the COVID-19 pandemic, it highlights the sense of precarity that has become a defining condition of the post-COVID world. Focusing on the US-based artist Carolyn Lazard, who lives with Crohn’s disease and multiple autoimmune conditions, and the Turner-prize winning British artist Jesse Darling, who developed neurological complications after giving birth, Smith adopts the term ‘crip aesthetics’ (Millett-Gallant 2018) to characterise the way sickness and disability have been politicised in contemporary art practice and activism.

Art history has traditionally approached illness, like madness, through the prism of romanticism: as a definitively individual experience, and as a marker of aesthetic sensitivity (Smith 2021, 2).^7^ Lazard and Darling pointedly refuse the role of the suffering artist as well as the (auto)biographical mode that has come to dominate both narrative medicine and much of the work across the medical humanities. They are not interested in sharing personal stories or representing disability in a more positive light. ‘In order to politicise sickness,’ says Lazard, ‘one has to depersonalise it first’ (quoted in Smith 2021, 9). Where narrative medicine privileges the individual patient’s voice, Darling and Lazard use sickness as a critical perspective on a collective experience of precarity: ‘a lens magnifying the structural fault lines that organise capitalist societies’ (Smith 2021, 5).

In ‘The limits of narrative: provocations for the medical humanities’, Angela Woods questions the emphasis on narrative models of meaning across the medical humanities and social sciences as well as in clinical training. Narrative, she observes, is assumed ‘to provide privileged access to the subjective experience of illness, and is frequently promoted as the primary vehicle through which the ill person can express her changing sense of self and identity’ (Woods 2011, 73). Using the philosopher Galen Strawson’s 2004 essay ‘Against Narrativity’ as a point of departure, Woods outlines the dangers of an uncritical reliance on narrative. Taking narrative as ‘*the* mode of human self-expression,’ she writes, ‘promotes a specific model of the self—as an agentic, authentic, autonomous storyteller’ (Woods 2011, 74). Disability scholar Eli Clare (2017) has drawn attention to the profoundly and problematically individualistic nature of modern medical practice, where ill-health is understood primarily as damage to a singular and independent body-mind, rather than as part of a broader ecology of social, political and economic factors. Recent attempts to depersonalise illness and disability (touched on briefly here) allow experiences of collective vulnerability to come into focus: by de-centring the personal story it becomes possible to attend to the wider social and cultural contexts of health.

Stella Bolaki observes a similar phenomenon in relation to artists’ books in which illness narratives are frequently characterised by multiplicity: as ‘narratives of community’ these visual and material ‘sickness stories’ often articulate a collective experience (Bolaki 2016, 1). Writing on self-portraits by artists with HIV and AIDS, Fiona Johnstone identifies a turn away from individualistic self-representation towards a visual language that recognises collective identities and the shared cultural contexts of health experiences: such works, Johnstone writes, ‘are never simply unmediated expressions of individual experience, but complex aesthetic and sociocultural entanglements’ ([Bibr R55][Bibr R56], 194). All three examples cited here—works by contemporary artists Lazard and Darling; artists’ books; and self-portraits made by artists with HIV and AIDS—suggest that visual language can articulate more richly nuanced and multifaceted forms of experience than written narrative alone.

The claim that artworks that draw on experiences of illness can be intellectually and aesthetically generative (as opposed to merely *representative* of personal experience) resonates with Tobin Siebers’ understanding of disability aesthetics. In his 2010 book of that title, Siebers makes it clear that he wants to go beyond the preoccupation with representation that had exercised disability activists and scholars in the preceding decades. That images can *disable* by perpetuating negative stereotypes is taken as given (Gartner and Joe 1987). Although *Disability Aesthetics* is, in part, a book about portrayals of disability in modern art, its more profound contribution is to begin to articulate a positive value for bodies and ways of being in the world that are more often stigmatised or defined in terms of lack, impairment, degeneration or deviation from an assumed norm. As an aesthetic value, disability offers a ‘critical resource for thinking about what a human being is’ (Siebers 2010, 3). Instead of the oppressive (as well as racist and ablest) ideal of physical perfection, disability presents us with infinite change, adaptation and variation, as well as ways of thinking about vulnerability, dependency and community. Ultimately, Siebers argues, an aesthetics of disability has the capacity to disrupt and enlarge our understanding of beauty, pleasure, and creativity (2010, 3).

Contemporary art organisations seldom engage explicitly with medical humanities or disability studies, but this is where some of the most important and compelling work in the medical humanities is currently being done (even if it rarely uses that label). Consider, for example, the exhibition *Lizzy Rose: Things I Have Learned the Hard Way* (various venues, Margate, UK, 31 March to 23 April 2023), which celebrated the life of British artist and disability activist Lizzy Rose (1988–2022).^8^ Rose lived with a severe form of Crohn’s disease: her multifaceted practice—including video, works on paper, photography, writing and curation—was shaped by, and gave form to, her experience of chronic illness. While the delicately pretty works on paper *Hospital Watercolour Club* (2014) explicitly reference time spent in medical spaces, other pieces take a more metaphorical, and often playful, approach to sickness: for example, Rose’s fantastically funny but also deadly serious video work *Sick Blue Sea* (2018), which is narrated by a teenage sperm whale suffering from gut pain and persistent nausea and vomiting; the whale’s stomach complaints are set off by ingesting dumped waste, drawing neat threads of connection between chronic ill-health and ecological damage. The exhibition programming included the live event *One Day I Will Feel My Power* (ICA London and streamed by Wysing Arts Center, April 2023). Curated by fellow artist Leah Clements, this event brought together video works and readings from a number of artists living with chronic health conditions, disability or neurodivergence, including Rose, Clements, RA Walden, Abi Palmer, Benedict Drew, Alice Hattrick and Carolyn Lazard.^9^

Rose also coproduced, with fellow artists Leah Clements and Alice Hattrick, the resource *Access Docs for Artists*, aimed at facilitating disabled artworkers in clearly outlining their access needs to arts organisations (which enjoy the status of working with disabled artists, but tend to be deeply ableist in their institutional structures).^10^ While art is often reduced to a biographical function (as we argued earlier), the work undertaken by Rose, Lazard, Clements, Hattrick and others, extends far beyond the strictly personal to address socioeconomic issues such as institutional infrastructures and inequities of access (to healthcare systems, art systems and other regimes of knowledge production and power). This work is essentially aesthetic: as Carolyn Lazard observes in a 2022 interview:

There’s this false notion that, if you make art which is tied to or interested in the world and in social conditions, you do not care about its aesthetic value. I’m deeply invested in beauty; I just don’t see the aesthetic value of art as conscripted exclusively to the relationship between the art viewer and the art object. It’s everywhere: in ideas, gestures, actions, care (Lazard and Bonhomme 2022).

Crip aesthetics (read here through the practices of contemporary ‘crip’ artists) approaches chronic illness and disability as an aesthetic experience, where aesthetics encompasses ‘affective relations between bodies’ (Siebers 2010, 1), beauty and politics. Medical humanities has much to learn from these artists’ interrogation of ableist presuppositions (including notions of bodily perfection, recovery and cure) as well as from artists’ practical strategies (such as the access rider) for more inclusive work environments; indeed, crip arts practices may be particularly well placed to respond to recent calls to build closer connections between medical humanities and disability studies (see Murray 2023).

The concept of care has animated a wide range of recent art-adjacent writing on health.^11^ The conjoining of art and aesthetics with care, as Lazard does in the quote above, can be usefully extended to consider the ways in which contemporary crip art practices situate themselves in relation to audiences. The speakers at the ICA event *One Day I Will Feel My Power* addressed a community of fellow disabled artists (as opposed to a universalising art-world version of the ‘general public’), effectively framing knowledge as a commodity produced by a specific community for the benefit of other members. In a similar way, groups like the US-based Canaries—a network for women and gender non-conforming people living with auto-immune conditions and other chronic illnesses—mobilise contemporary art strategies and structures to communicate with an audience of fellow sick people.^12^ When Taraneh Fazeli, a curator and a member of the Canaries, secured a residency supported by the Museum of Fine Art, Houston’s Core Program to develop the project *Sick Time, Sleepy Time, Crip Time,* they invited their ‘fellow canaries in the coal mine’ (Fazeli 2016) to contribute.^13^ The resulting coproduced broadsheet, *Notes for the Waiting Room* (2016), was printed and distributed in art and medical contexts, including doctors’ waiting rooms.^14^ A toolkit based on collective lived experience, *Notes for the Waiting Room* sits somewhere between self-help and critical theory, mixing practical tips for living with, for example, Crohns Disease with a recommended reading list that encompasses theory (Mel Y. Chen, Susan Sontag, Georges Didi-Huberman, Alison Kafer and Jean-Luc Nancy), history, illness memoir and more. Although the project’s most visible output was the exhibition *Sick Time, Sleepy Time, Crip Time* (The EFA Project Space 30 March—13 May 2016, then touring), Fazeli was explicit that the show was just an ‘access point’ beyond which was ‘a vast rhizomatic underbelly of workshops, performances and group work that was intentionally not available to all’. (Clements, Fazeli, and MacBride 2021). While making use of art-world spaces (understood here both as physical site and conceptual framing), it is not unusual for the core activities of this kind of art work to remain invisible to all but a particular group.

A similar strategy of ‘aesthetic refusal’ (Molesworth 2018, 171–172) informs American artist Simone Leigh’s work *The Waiting Room*, which was installed at the New Museum in New York in 2016. A tribute to the black woman Esmin Elizabeth Green who died in a New York emergency room while waiting to see a doctor, *The Waiting Room* extended Leigh’s earlier investigations into histories of black healthcare including the project *Free People’s Medical Clinic* (2014). As recalled by the curator Helen Molesworth, *The Waiting Room* was ‘not a particularly visual event’ (2018, 166): on Molesworth’s visit, it consisted of a relatively empty gallery space whose focal point was a large cabinet lined with glass jars. This, Molesworth acknowledged, ‘suggested that the real work lay elsewhere, namely, in the workshops on such topics as complementary medicine, folk healing traditions, acupuncture and meditation’ (2018, 166).^15^ Aimed at a primary audience of black women, the majority of the exhibition’s workshops were closed to a general public and were not documented for the archive. Initially disgruntled that she (a white woman) was unable to access the work, Molesworth came to appreciate the significance of this tactic: ‘Leigh’s work makes it plain that I can’t enter into its field of knowledge, when it suggests that my capacity for sight and empathy and knowledge is bounded and limited’ (2018, 172). The recognition that some forms of knowledge are not universally accessible poses an interesting provocation for the medical humanities, with implications for ongoing debates around the role of lived experience in relation to research and knowledge production.

We have chosen to highlight these specific examples of contemporary art history writing and art practice because, although they do not explicitly engage with the research agendas of critical medical humanities, they have much to offer medical humanities as a field.^16^ These practices can facilitate critical thinking about the sociopolitical contexts of illness; offer a useful corrective to the assumptions about selfhood and individual subjectivity that have often dominated narrative medicine and prompt us to reconceptualise experiences of chronic illness and disability as intellectually and aesthetically generative (while resisting presumptively ableist linear narratives of recovery or cure). They also offer methodological strategies for coproduction, collaboration and the creation of inclusive work environments and raise important questions about the assumed audiences for medical humanities research.

## New models of collaborative entanglement

This section explores three recent art history-led medical humanities projects that have responded, either implicitly or explicitly, to calls for a more ‘entangled’ critical medical humanities: *Art Hx: Visual and Medical Legacies of British Colonialism; Visualizing the Virus; and Confabulations: Art History, Art Practice, Critical Medical Humanities*. All three were devised and launched during the COVID-19 pandemic (although only one, *Visualizing the Virus*, responds to COVID-19 directly); all mobilise the digital space to reach beyond an audience of disciplinary specialists.

*Art Hx: Visual and Medical Legacies of British Colonialism* (launched 2020) is a digital art history project exploring the intersections of art, medicine and race in the British Empire.^17^ The project was directly shaped by the professional experiences of the PI, Anna Arabindan-Kesson, who had trained and worked as a registered nurse (in New Zealand, Australia and the UK) before completing a PhD in art history and establishing an academic career; Arabindan-Kesson credits the First Nations women that she was trained by and worked with for teaching her how to think critically about the inequalities of a healthcare system that was failing to addresses the health disparities of First Nations Communities and people of colour. Of particular significance for this article, Arabindan-Kesson compares the method of ‘critical seeing’ modelled by these women as akin to the disciplinary tools used by art historians.^18^ The *Art Hx* website consists of constellations of images and short-form texts produced by team members, fellows (contemporary artists and early career academics) and affiliates, arranged around three thematic frameworks (Cultivating Care, Pathologies of Difference and Medicalized Spaces). To give a sense of the thematic and material scope of the project, recently published content includes a text on black women healers and modern gynaecology that responds to an image in an 1870 illustrated medical monograph (Bonhomme 2022); a documentation of eighteenth-century representations of Caribbean-identified medicinal plants hosted in collections around the globe (Reid 2022); and an essay locating an early twentieth-century contraceptive cap stamped with the word ‘PRORACE’ within broader colonial narratives of healthcare (Saggar 2023). Supported by regular online speaker events and symposia, the *Art Hx* enterprise as a whole suggests a lively and still-gestating research area that is likely to result in field-defining publications and new projects in years to come.

*Visualizing the Virus* (launched 2021) is an award-winning digital humanities project founded and led by Sria Chatterjee, an art historian and environmental humanities scholar who is also a powerful advocate for the value of using the ‘tools’ and methods of art history for addressing the most pressing concerns of our time.^19^ Focussing on connection—which, as Chatterjee notes, is ‘crucial to the work of making visible how contagion is contingent on various social, political and economic processes’ (2022)—the purpose of the project is to understand the practices (both historical and contemporary) that have made viruses visible as well as the ways in which the virus has brought other issues (health inequalities, xenophobia, vaccine hesitancy) to matter. The architecture of the online platform gives graphic form to these connections: individual contributions by authors working in different disciplines are represented as small colourful discs orbiting themed circular clusters with titles like ‘What COVID-19 gave us: citizen agency in East Africa’; ‘Discards of COVID-19’ and ‘Pandemic Dwelling’. Many contributions are accessible short-form summaries of already-published, peer-reviewed texts: the effect is akin to an interdisciplinary market place for the exchange and cross-fertilisation of ideas, activated through visualising (explicitly positioned by Chatterjee (2022) ‘as a verb to mobilize a method’).

*Confabulations: Art History, Art Practice, Critical Medical Humanities* is an online seminar series (2021–2023) and book project, developed with the aim of making explicit the contributions that artists and art historians can make to developments in critical medical humanities, while also seeking to expand the boundaries of medically orientated art history beyond existing scholarship on, for example, anatomical imagery, clinical portraiture and self-representations of experiences of illness.^20^ Recognising that many artists work on issues relating to health and medicalised bodies without explicitly referencing medical humanities and that many art historians work on aspects of medical history without positioning themselves as medical humanities scholars, *Confabulations* called on artists and art historians who were ‘medical humanities curious’, as well as those already identifying with the field. With a title inspired by Hartman (2008), Berger (2016), and psychological processes of imagination, the series foregrounded methodological risk, innovation and creative boundary crossing and focused on establishing dialogue between artists and academics doing medical humanities research. The seminar series (and forthcoming book) explored a diverse range of themes that included different disciplinary approaches to ‘lived experience’; gendered experiences of healthcare; visually engaged activist health practices; critical histories of art therapy; mental health histories; contemporary art and biomedical imaging; COVID-19; the medicalised body; humour and art; and the embodied dynamics of horizontality. Perhaps most significantly, the series demonstrated the value of art-led methods for medical humanities research.

*Confabulations* continues the work of two further interdisciplinary medical humanities and visual and material culture projects led by *Confabulation’s* codirectors. The first, *Thinking Through Things: Object Encounters in the Medical Humanities* (2019–2021) asked what might be gained by ‘doing’ medical humanities through objects, images and artworks and supported a programme of activities designed to stimulate interdisciplinary dialogue around the holdings of Wellcome Collection in London.^21^ The second denotes a number of interconnected curatorial activities led by Allison Morehead with the aim of bringing together art history, medical history and medical humanities through creative methods, including the exhibition *Edvard Munch and the Medicalization of Modern Life* (27 June to 21 September 2025, Munchmuseet, Oslo).

We end this section with a snapshot of how academic institutions are responding to and shaping the emerging field. In 2021, the Australian National University ran a symposium titled ‘Visualising the Medical Humanities’; the call for papers confidently declared that ‘The Visual Medical Humanities […] has been recognised as a major area for research and teaching innovation in the new critical Medical Humanities.’^22^ In Germany, the Institute for Medical & Health Humanities and Artistic Research, established in 2022, advocates for the value of artistic research in transdisciplinary health humanities work, organising regular online events and other activities.^23^ In 2023, Durham University (UK) announced that a Visual and Material Lab would form one of six methods-led research strands in the newly launched Discovery Research Platform for Medical Humanities (2023–2030).^24^ In the USA, the art historian Tanya Sheehan directs a Public Humanistic Inquiry Lab (2021–2024) at Colby College that critically explores the connections between medicine and race.^25^ While much of this activity has not yet translated into a formal peer-reviewed literature, it hints at a proliferating subfield of (visual) medical humanities research that will enjoy significant future growth.

## Closing thoughts: what can art history do?

What does medical humanities miss by not engaging more extensively with art history and contemporary arts practices? Why, at this juncture, is a more visually attuned medical humanities necessary? And what can art historians do to better articulate the contributions that they can make? Before we offer some closing thoughts, it is worth returning briefly to Fitzgerald and Callard’s analysis of the state of the field, and in particular to calls for a ‘critical’ medical humanities. Instead of being ‘*useful* to biomedicine’, the medical humanities has the potential to open up new spaces and modes of enquiry. How, they ask:

might the methodological and intellectual legacies of the humanities intervene more *consequentially* in the clinical research practices of biomedicine—situating accounts of illness, suffering, intervention and cure in a much thicker attention to the social, human and cultural contexts in which those accounts, as well as the bodies to which they attend, become both thinkable and visible? (Fitzgerald and Callard 2016, 35)

We have suggested in this article that art is one of the significant contexts in which illness and health, medical intervention and cure become thinkable and visible, often in expansive and sometime surprising ways. Art historians are trained to approach their objects of study as cultural artefacts that *do* things (both in the present moment and in the past)—as commodities, as cultural capital, as bearers of ideology, taste and feeling, as ‘working objects’ that embody epistemic virtues and train the eye (Daston and Galison 2007, 19, 22). And although art history has foregrounded certain kinds of objects (and called them art), its disciplinary practices—an evolving and often contested set of skills, debates, approaches and conventions—allow us to think critically about non-art objects, environments and ideas.

A sustained consideration of art and visual culture offers rich opportunities for advancing research on human health experience. Art historians take art and visual culture seriously; we advocate that scholars working in the critical medical humanities should do likewise. At the same time, we suggest that art historians wishing to develop a closer dialogue with medical humanities scholarship need to be better at articulating the value of their discipline to a non-discipline specific audience; this might involve publishing in non-specialist journals, or engaging with interdisciplinary projects and platforms (like those outlined in the penultimate section of this article) as a necessary first step to successfully ‘entangling’ art history’s methods and materials within a wider flourishing field.

In this article, we have traced the formation of the nascent field of visual medical humanities over the past decade, identifying the dominant intellectual, methodological and disciplinary histories that have shaped the ways in which medical humanities scholarship has engaged with art and visual culture to date and proposed a number of new directions for research beyond the prevailing tropes of the anatomy theatre, the clinical encounter and the biographical narrative. By arguing for the vital importance of attending to the critical complexities of art and visual culture, we hope to enrich existing debates about health, illness and culture and provoke a new wave of visually engaged medical humanities scholarship.

## Data Availability

No data are available.

## Footnotes

For a general introduction to visual culture see Mirzoeff (2013). For an overview of debates around the emergence of visual culture in relation to art history, see Dikovitskaya (2006). For a recent discussion of visual culture as an active critical practice that renders phenomena visible or invisible see Mirzoeff (2023, 1–4), 1–4.

Each of these areas has their own, often extensive, literature: the following texts are suggested as starting points for readers that may be less familiar with the field(s). On medical imaging, see Cartwright (1995); Kevles 1997Kevles (1997)Kevles (1997); van Dijck 2005van Dijck, José (2005) van Dijck (2005); Waldby (2000); Casini (2021). For an excellent article on public health posters, see Cooter and Stein (2007). For an edited volume on public health and visual culture, see Serlin (2010). On film and television in relation to public understandings of health, see Bonah and Laukötter (2020) and Ledin and Weil (2023)Ledin and Weil 2022. On the history of art therapy in Britain, see Hogan (2001)Hogan 2001. On social media, selfies and ill-health see Tembeck (2016). For a helpful overview of a range of medical museums within a single volume, see Hallam and Alberti (2013)Hallam and Alberti 3013. For a recent history of arts-in-health in Britain, see Williams (2023). For a critical re-appraisal of art therapy in relation to art history, see Sheehan and Hudson (2024) Sheehan (2024). The Graphic Medicine website (https://www.graphicmedicine.org) provides an excellent starting point for those interested in Graphic Medicine, as does Czerwiec *et al* (2015))Czerwiec et. al. 2015.

For two interesting examples of early contemporary art writing on the pandemic, see Preciado (2020) and Vishmidt (2020). For an art historical account of the parallels between COVID-19 and previous pandemics, see Morton and Akehurst (2023). On the visual rhetoric of healthcare evidence during the COVID-19 pandemic, see Sonnevend (2020) and Jones (2023).

A brief survey of this kind presents an obvious challenge in determining what does and does not ‘“count’” as art history. For example, chapters on Graphic Medicine are routinely included in such readers. Although a visually orientated genre, graphic medicine has more typically been addressed by disciplines other than art history; for this reason, we have bracketed it as outside the scope of this article.

Key examples of art history and anatomy crossover projects include the exhibitions The Quick and the Dead curated by Deanna Petherbridge in 1998; Spectacular Bodies curated by Martin Kemp and Marina Wallace in 2000; Anatomy Acts curated by Dawn Kemp and Andrew Patrizio in 2006; Dream Anatomy curated by Michael Sappol in 2006; and more recently, an interactive feature in British Art Studies on Victorian anatomical atlases guest edited by Keren Hammerschlag. See Petherbridge and Jordanova (1997)Petherbridge and Jordanona 1997; Kemp and Wallace (2000)Kemp and Wallace 2000; Patrizio and Kemp (2006); Sappol (2006); Hammerschlag 2021[Bibr R44][Bibr R45]. Books on the long history of anatomical display and illustration include Maerker (2011); Stephens (2011); Hallam (2016); Wils, de Bont, and Au (2017)Wils, de Bont and Au 2017; Hartnell (2018); Graciano (2019). One might also consider the rich literature on biomedical sci-art collaborations (Anker and Nelkin 2004; Ede 2000; Nelkin 2007) and bioart ([Bibr R61][Bibr R62] and 2007; Mitchell 2010; Myers 2015).

Hammerschlag and Ruiz-Gómez have focused on the symbiotic relationship between artistic and medical ‘“looking’” in subsequent publications (Hammerschlag 2016; 2021[Bibr R44][Bibr R45]; [Bibr R93][Bibr R94][Bibr R95] 2019; 2021; ). The close relationship between clinical and artistic vision – —including the haptic and tactile qualities of both – —areis also explored in Allison Morehead’s catalogue essay on the nineteenth-century artists August Strindberg and Edvard Munch (2019).

For a foundational text on the modern trope of the (male) artist as tortured genius see Pollock (1980). On the cultural archetypes and creative possibilities associated with female madness in the long eighteenth century, see [Bibr R52][Bibr R53] and Jamieson 2022.

Lizzy Rose: Things I Have LearnedLearned the Hard Way, various venues, Margate, UK, 31 March to 23 April 2023.

One Day I Will Feel My Power (ICA London and streamed by Wysing Arts Center, April 2023). A recording of this event is available at https://www.youtube.com/watch?v=8NFkD0D06XQ. Accessed 26 May 2023.

For more on Rose’s work, see Wallis (2023, 35–36), 35–36.

See for example the section ‘“Self Caring’” in Rodríguez Muñoz (2020, 124–153), 124–153); Jagoe and Kivland (2020); Blackshaw and Kivland (2021). On Care Aesthetics, see Thompson (2023).

For more on the Canaries, see https://digigiid.ee/en/exhibitions/disarming-language/canaries [a. Accessed 17 January 2024]..

See also Bise (2016)Bise 2016 for a critique of the assumptions underpinning ‘“Sick Time, Sleepy Time, Crip Time’”.

A PDF copy of Notes for the Waiting Room can accessed online at: https://static1.squarespace.com/static/537c9eb6e4b0ca838c2f4f52/t/5eaafc3bc2609667f6a9ae23/1588264038420/NFTWR_LincoV5.pdf. Accessed 25 January 2024.

The ‘“hands-on’” nature of many of these activities resonates with Sieber’s understanding of aesthetics as ‘“relations between bodies’” (2010), as well as James Thompson’s articulation of ‘“care aesthetics’” (2023).

This observation is not intended to deny the existence of contemporary artists who are already engaging directly with medical humanities: see Johnstone (2023b) for a fuller discussion of these practices.

https://artandcolonialmedicine.com. Accessed 26 January 2024.

Taken from https://artandcolonialmedicine.com/team/. Accessed 25 January 2024.

https://visualizingthevirus.com. Accessed 26 January 2024.

https://confabulationsdotorg.wordpress.com. Accessed 26 January 2024. The edited volume Art and the Critical Medical Humanities, is forthcoming with Bloomsbury in their Critical Interventions in Medical and Health Humanities series.

http://nnmh.org.uk/thinking-through-things/. Accessed 30 January 2024.

https://healthmedicalhumanities.net.au/visualising-the-medical-humanities-a-symposium-anu-22-july/. Accessed 25 January 2024. The symposium was supported by a four4 year ANU Futures Scheme Award made to Keren Hammerschlag to develop the Visual Medical Humanities at the ANU; the same award also supported a PhD studentship in Visual Medical Humanities. For a summary of the symposium see Blake (2021)Blake 2021.

https://www.imhar.net. Accessed 25 January 2024.

https://www.durham.ac.uk/research/institutes-and-centres/discovery-research-platform-for-medical-humanities/. Accessed 25 January 2024.

https://web.colby.edu/medrace/. Accessed 25 January 2024.
